# Two Isoforms of *Yersinia pestis* Plasminogen Activator Pla: Intraspecies Distribution, Intrinsic Disorder Propensity, and Contribution to Virulence

**DOI:** 10.1371/journal.pone.0168089

**Published:** 2016-12-09

**Authors:** Svetlana V. Dentovskaya, Mikhail E. Platonov, Tat’yana E. Svetoch, Pavel Kh. Kopylov, Tat’yana I. Kombarova, Sergey A. Ivanov, Rima Z. Shaikhutdinova, Lyubov’ V. Kolombet, Sadhana Chauhan, Vitaly G. Ablamunits, Vladimir L. Motin, Vladimir N. Uversky, Andrey P. Anisimov

**Affiliations:** 1 State Research Center for Applied Microbiology and Biotechnology, Obolensk, Moscow Region, Russian Federation; 2 Department of Pathology, University of Texas Medical Branch, Galveston, Texas, United States of America; 3 Department of Microbiology & Immunology, University of Texas Medical Branch, Galveston, Texas, United States of America; 4 Saint Petersburg Medical Pediatric University, Saint Petersburg, Russia; 5 Department of Molecular Medicine and Byrd Alzheimer's Research Institute, Morsani College of Medicine, University of South Florida, Tampa, Florida, United States of America; 6 Institute for Biological Instrumentation, Russian Academy of Sciences, Pushchino, Moscow Region, Russian Federation; University of Toledo College of Medicine and Life Sciences, UNITED STATES

## Abstract

It has been shown previously that several endemic *Y*. *pestis* isolates with limited virulence contained the I259 isoform of the outer membrane protease Pla, while the epidemic highly virulent strains possessed only the T259 Pla isoform. Our sequence analysis of the *pla* gene from 118 *Y*. *pestis* subsp. *microtus* strains revealed that the I259 isoform was present exclusively in the endemic strains providing a convictive evidence of more ancestral origin of this isoform. Analysis of the effects of the I259T polymorphism on the intrinsic disorder propensity of Pla revealed that the I259T mutation slightly increases the intrinsic disorder propensity of the C-terminal tail of Pla and makes this protein slightly more prone for disorder-based protein-protein interactions, suggesting that the T259 Pla could be functionally more active than the I259 Pla. This assumption was proven experimentally by assessing the coagulase and fibrinolytic activities of the two Pla isoforms in human plasma, as well as in a direct fluorometric assay with the Pla peptide substrate. The virulence testing of Pla-negative or expressing the I259 and T259 Pla isoforms *Y*. *pestis* subsp. *microtus* and subsp. *pestis* strains did not reveal any significant difference in LD_50_ values and dose-dependent survival assays between them by using a subcutaneous route of challenge of mice and guinea pigs or intradermal challenge of mice. However, a significant decrease in time-to-death was observed in animals infected with the epidemic T259 Pla-producing strains as compared to the parent Pla-negative variants. Survival curves of the endemic I259 Pla^+^ strains fit between them, but significant difference in mean time to death post infection between the Pla^−^strains and their I259 Pla^+^ variants could be seen only in the isogenic set of subsp. *pestis* strains. These findings suggest an essential role for the outer membrane protease Pla evolution in *Y*. *pestis* bubonic infection exacerbation that is necessary for intensification of epidemic process from endemic natural focality with sporadic cases in men to rapidly expanding epizootics followed by human epidemic outbreaks, local epidemics or even pandemics.

## 1. Introduction

The plague pathogen, *Yersinia pestis*, was the reason of the death of millions of people worldwide. This Gram-negative bacterium causes a flea-borne zoonosis in populations of wild rodents inhabiting natural plague foci [1–3]. The efficacy of the pathogen transmission, acute course, and lethal outcome of this infection are supported by numerous bacterial transmission and/or pathogenicity factors [4–7]. One of them, plasminogen activator Pla, is a representative of omptins, a family of enterobacterial outer membrane proteases that are responsible for colonization of the specific organs or even infection generalization as a result of successful circumvention of the host innate immunity [8, 9].

Pla is encoded by the 9.5 kb *Y*. *pestis* plasmid pPCP [10]. Pla expression is associated with a marked ability to colonize the viscera and thus cause lethal infection upon administration by peripheral routes of infection, such as intradermal (i.d.) or subcutaneous (s.c.) [11]. The Pla activity is not required to initiate lethal disease by intravenous injection (i.v.), which provides immediate access to fixed macrophages lining the capillary beds of the liver and spleen [12]. This relationship was verified with isogenic *pla* mutants of epidemic *Y*. *pestis* strains KIM and CO92, which showed up to 10^6^ logs of reduced virulence by the s.c. route, in comparison with the i.v. route, in a murine model of plague [11, 13]. Since in nature plague is transmitted by fleas, *Y*. *pestis* exhibits a remarkably efficient ability to spread from the peripheral site of the flea bite to the regional lymph node, after which it multiplies and further invades the circulation. The major role in this process has been attributed to Pla because this protease resembles mammalian plasminogen activators in function and converts plasminogen to plasmin by limited proteolysis [14], possibly leading to clarification of fibrin deposits that could hinder bacterial migration in the circulation [15].

In sum several studies performed on the strains belonging to bv. medievalis/SNP-type 2.MED and bv. orientalis/1.ORI indicated that Pla is required for full virulence in subcutaneous/intradermal infection [11, 13, 16], is necessary for the development of the bubonic *vs*. septicemic form of the disease [11, 13, 16], and is essential for *Y*. *pestis* to cause primary pneumonic plague [17]. Also, it was shown that Pla can directly inactivate major plasmin inhibitor α_2_-antiplasmin [18, 19] and mediate adhesion to eukaryotic cells (extracellular matrices and basement membranes) which invasive bacteria must penetrate in order to reach the circulation [20, 21]. In addition to its role in adhesion, invasion and tissue damage, Pla has been reported to cleave complement component C3 [11, 22], to possess weak coagulase activity [15], and to mediate the proteolysis of *Yersinia* virulence factors Yops [23]. Recently it was shown that Pla provides proteolytic processing of the *Y*. *pestis* autotransporters YapG and YapE [24, 25], cleaves and inactivates mammalian thrombin-activatable fibrinolysis inhibitor (TAFI) [26], plasminogen activator inhibitor 1 (PAI-1) [27, 28], endogenous anticoagulant tissue factor pathway inhibitor (TFPI) [29], host factor peroxiredoxin 6 [30], and degrades the apoptotic signaling molecule Fas ligand (FasL) [31]. Finally, murine C-type lectin receptor DEC-205 (CD205) expressed on both alveolar macrophages and pulmonary dendritic cells is a receptor for Pla of *Y*. *pestis*, and their interaction plays a key role in promoting bacterial dissemination [32].

However, other *Y*. *pestis* strains lacking *pla* are still highly virulent when administered by subcutaneous and/or respiratory routes [1, 13, 33, 34]. More recently, it was shown that Pla is not required for bacterial translocation to the lymph node and that a Pla^−^*Y*. *pestis* caused the same extensive histological lesions as the wild type strain [35]. It was suggested, that the primary action of Pla is to protect bacteria from destruction rather than to alter the tissue environment to favor *Y*. *pestis* propagation in the host [36].

This pathogenicity factor was absent in representatives of the *Y*. *pestis* subsp. *microtus* bv. caucasica/SNP-type 0.PE2 [37], while an ancestral Pla isoform (I259) with characteristics similar to the properties of omptins from less virulent enterobacteria has been found in three representatives [38, 39] of *Y*. *pestis* subsp. *microtus* (SNP-types 0.PE3 and 0.PE4), which are, as a rule, avirulent to guinea pigs and humans [1]. The “modern” isoform of Pla (T259) with an increased protease activity was found in *Y*. *pestis* subsp. *pestis* strains that are highly virulent for humans [38]. It has been recently shown that Pla I259T modification was not required to cause pneumonic plague in the murine intranasal model (surrogate for respiratory challenge), as both isoforms were sufficient to induce primary pneumonia. Nevertheless, the “modern” isoform of Pla (T259) more efficiently induced the invasive infection associated with bubonic form of the disease in mice [39].

The contradictory data on the role of Pla in *Y*. *pestis* virulence might result not only from different strain backgrounds but also from methodological differences in the generation of such isogenic strains. It was previously suggested [40] that transfers of genetic information are more frequent in atypical forms of certain bacteria. In this case, one should speak not of a decreased virulence of the cells, but of a change in the composition of the population, caused by an accumulation (selection) of recombinants with an initially low virulence or entirely avirulent. The selection of clones that have retained virulence at the level of the wild-type parent strains requires an animal passage. In evaluating the virulence of cultures of agents of infectious diseases subjected to experimental manipulations or stored for long periods under laboratory conditions, a preliminary stage of animal passage is necessary to purify the microbial population from avirulent segregants [41].

In this study, we provide an evidence that the I259 isoform of Pla is present exclusively in the endemic *Y*. *pestis* strains that is supported by sequencing the *pla* genes from 118 strains of *Y*. *pestis* belonging to seven of eight biovars of subsp. *microtus* [37], and circulating on the territory of the former Soviet Union and Mongolia. Our computational analysis of intrinsic disorder propensity of Pla predicted that the Pla isoform found in *Y*. *pestis* epidemic strains should be more functionally efficient than that of the endemic strains, and this hypothesis was confirmed experimentally. Finally, we evaluated the virulence properties of *Y*. *pestis* subsp. *microtus* and subsp. *pestis* strains expressing the Pla isoforms. We found no significant difference in LD_50_ values and dose-dependent survival assays between the strains with the I259 and T259 Pla by using a subcutaneous route of challenge of mice and guinea pigs, or intradermal challenge of mice. A significant difference could be seen in mean time to death post infection between the Pla^−^strains and their T259 Pla^+^ variants in the both sets of isogenic strains. Survival curves of the I259 Pla^+^ strains fit between them, but significant difference in mean time to death post infection between the Pla^−^strains and their I259 Pla^+^ variants could be seen only in the isogenic set of subsp. *pestis* strains.

## 2. Materials and Methods

### Bacterial strains and growth conditions

*Y*. *pestis* intraspecies classification used in this study corresponds to the International Codex of Bacterial Nomenclature [37, 42, 43]. In total, we used 118 strains of *Y*. *pestis* representing seven out of eight biovars, such as caucasica (78), altaica (17), qinghaiensis (2), xilingolensis (3), hissarica (4), talassica (4), and ulegeica (10) belonging to subsp. *microtus* [37], as well as several strains of the main subsp. *pestis* of the biovars antiqua, medievalis, and orientalis. Characteristics of the strains used for testing of their fibrinolytic and coagulase activities or for virulence studies are shown in Table 1.

**Table 1:** Strains and plasmids used in this study for testing Pla activity and virulence experiments.

*Y*. *pestis* strain or plasmid	Description	Source/reference
**subsp. *pestis* bv. *antiqua***		
231pPst^-^	pPst^-^ derivative of the wild type strain 231; universally virulent	SCPMO* [44]
231pPst^-^pkPI-3455	As 231pPst^-^, but harboring plasmid pkPI-3455	The authors' collection
231pPst^-^pkPEV	As 231pPst^-^, but harboring plasmid pkPEV	The authors' collection
**subsp. *pestis* bv. medievalis**		
358pCD1^-^	pCD1^-^ derivative of the universally virulent wild type strain 358; avirulent	SCPMO
358pCD1^-^pPst^-^	pPst^-^ derivative of the strain 358pCD1^-^; avirulent	SCPMO
358pCD1^-^pPst^-^pkPI-3455	As 358pCD1^-^pPst^-^, but harboring plasmid pkPI-3455	The authors' collection
358pCD1^-^pPst^-^pkPEV	As 358pCD1^-^pPst^-^, but harboring plasmid pkPEV	The authors' collection
KIM D27	Pigmentation negative derivative harboring plasmids pMT1, pCD1, pPCP1	Brubaker’s collection
KIM D47	Pigmentation negative deriative harboring plasmid pMT1	Brubaker’s collection
KIM D47/ pPCP1Km	KIM D47 harboring plasmid pPCP1Km	The authors' collection
KIM D47 /pPCP1KmCh	KIM D47 harboring plasmid pPCP1KmCh	The authors' collection
**subsp. *pestis* bv. orientalis**		
KM217	pMT1^-^pPst^-^ derivative of the vaccine strain EV line NIIEG; avirulent	SCPMO
KM217pkPI-3455	As KM217, but harboring plasmid pkPI-3455	The authors' collection
KM217pkPEV	As KM217, but harboring plasmid pkPEV	The authors' collection
**subsp. *microtus* bv. caucasica** (0.PE2)		
C-267	Naturally pPst^-^ strain virulent for voles and mice	SCPMO
C-267pkPI-3455	As C-267, but harboring plasmid pkPI-3455	The authors' collection
C-267pkPEV	As C-267, but harboring plasmid pkPEV	The authors' collection
C-376pCD1^-^	pCD1^-^ derivative of naturally pPst^-^ strain C-376 virulent for voles and mice; avirulent	The authors' collection
C-376pCD1^-^pkPI-3455	As C-376pCD1^-^, but harboring plasmid pkPI-3455	The authors' collection
C-376pCD1^-^pkPEV	As C-376pCD1^-^, but harboring plasmid pkPEV	The authors' collection
C-585pCD1^-^	pCD1^-^ derivative of naturally pPst^-^ strain C-585 virulent for voles and mice; avirulent	The authors' collection
C-585pCD1^-^pkPI-3455	As C-585pCD1^-^, but harboring plasmid pkPI-3455	The authors' collection
C-585pCD1^-^pkPEV	As C-585pCD1^-^, but harboring plasmid pkPEV	The authors' collection
C-824pCD1^-^	pCD1^-^ derivative of naturally pPst^-^ strain C-824 virulent for voles and mice; avirulent	The authors' collection
C-824pCD1^-^pkPI-3455	As C-824pCD1^-^, but harboring plasmid pkPI-3455	The authors' collection
C-824pCD1^-^pkPEV	As C-824pCD1^-^, but harboring plasmid pkPEV	The authors' collection
**Plasmids**		
pkPI-3455	PstI fragment of plasmid pUC4K (X06404.1) containing the Km^R^ cassette was introduced without gene disruption into the PstI site of plasmid pPst from *Y*. *pestis* subsp. *microtus* bv. altaica strain I-3455	The authors' collection
pkPEV	PstI fragment of plasmid pUC4K containing the Km^R^ cassette was introduced without gene disruption into the PstI site of plasmid pPst from *Y*. *pestis* subsp. *pestis* bv. orientalis strain EV line NIIEG (GenBank accession no. JBOL00000000.1)	The authors' collection
pPCP1Km	PstI fragment of plasmid pUC4K containing the Km^R^ cassette was introduced without gene disruption into the PstI site of plasmid pPCP1 from *Y*. *pestis* subs. *pestis* bv. medievalis strain KIM D27	The authors' collection
pPCP1KmCh	Plasmid pPCP1Km with point mutation resulting in Pla I259T modification	The authors' collection

* The State Collection of Pathogenic Microbes and Cell Cultures on the base of the State Research Center for Applied Microbiology and Biotechnology (“SCPM-Obolensk”; http://obolensk.org/center/state-collection.htm).

Bacterial cultures were initiated from the lyophilized stocks. *Y*. *pestis* strains underwent two passages in mice prior to be used for animal challenge.

*Y*. *pestis* strains were grown at 28°C for 48 h on brain heart infusion (BHI; HiMedia Laboratories) supplemented with 2% agar at pH 7.2. For testing Pla activity with the fluorescent substrate, bacteria were grown on Heart Infusion Broth (HIB) agar plates at 26°C or 37°C for 24 h.

All handling of samples containing live wild-type *Y*. *pestis* isolates and their virulent derivatives was performed in a select agent authorized BSL3 facility under protocols approved by the State Research Center for Applied Microbiology and Biotechnology Institutional Biosafety Committee.

### Determination of nucleotide sequence of *pla* genes

The nucleotide sequence of each *pla* gene was determined by the direct sequencing of the PCR fragment obtained after amplification of the *pla* gene of the corresponding strain. The primers pla-F1 (5’- TAATATGTTTTCGTTCATGC -3’) and pla-R1 (5’- CGCTAGGGGAGGATGAAAAG -3’), both flanking the *pla* gene, were located within the YPPCP1.06 and YPPCP1.08c genes, respectively. These sequences of the *pla* gene were compared to the corresponding sequences of two alleles of this gene from other *Y*. *pestis* strains available in the GenBank (accession nos. AE017046.1 and KM880025.1). *Y*. *pestis* strains and DNA isolates were obtained either from the State Research Center for Applied Microbiology and Biotechnology (SRCAMB), Obolensk (Moscow Region, Russia), or kindly provided by Prof. S.V. Balakhonov (Antiplague Research Institute of Siberia and Far East, Irkutsk, Russia).

### Measurement of the Pla protein expression

The level of production of the Pla isoforms was assessed from the *Y*. *pestis* whole-cell lysates. The whole-cell lysates were prepared by mixing bacteria (10^9^ colony forming units (CFU)/ml) with half of the volume of SDS-PAGE loading buffer, and boiled for 10 min. Samples were loaded into a 12.5% (wt/vol) SDS-PAGE gel followed by electrophoresis and transfer on a nitrocellulose membrane for detection with 3F3 monoclonal antibody (MAb) to Pla (Pushchino Branch of the M.M. Shemyakin & Yu.A. Ovchinnikov Institute of Bioorganic Chemistry of the Russian Academy of Sciences) used at 1:4,000 dilution. Then the membrane was incubated with the horseradish peroxidase-conjugated anti-mouse immunoglobulin G (GE Healthcare) secondary antibody, and developed with 3,3′-diaminobenzidine. The area of bands was measured using Gel Analyzer software.

### Analysis of the coagulase and fibrinolytic activities

Coagulase and fibrinolytic activities were determined as previously described [45]. A pool of normal human plasma (NHP) was obtained from ten naïve healthy volunteers. A positive coagulase test (++) was represented by a solid clot; a positive test (+) by any degree of incomplete clotting (from a loose clot to a solid clot in liquid plasma); a negative test (−) by the absence of clotting. A positive fibrinolysis test (++) corresponded to a complete clot lysis; a positive test (+) by any degree of lysis; a negative test (−) by a solid clot.

### Analysis of the Pla enzymatic activity using a fluorometric assay

The Pla activity was evaluated using a fluorometric assay as previously described [46, 47]. *Y*. *pestis* strains were grown on HIB agar at 26°C for 36 h, replated to fresh HIB, and incubated at either 26°C or 37°C for 24 h. Bacteria were harvested from the plates, suspended in PBS and adjusted to optical densities (OD_600_) of 0.1 (5 × 10^7^ cfu/ml) and 0.05 (2.5 × 10^7^ cfu/ml) for the cultures grown at 26°C and 37°C, respectively. The bacterial titers were verified by plating the suspensions on HIB agar in 10-fold dilutions. The assay was conducted with 50 μl of bacterial suspension containing 2.5 μg of the fluorescently labeled hexapeptide substrate DABCYL-Arg-Arg-Ile-Asn-Arg-Glu (EDANS)-NH_2_ [48] in the black 96-well microplate (Costar Corning Inc., Corning, NY) in quadruplicates. The kinetics of substrate cleavage by the Pla expressed on the surface of *Y*. *pestis* cells was measured every 10 min for 6 h at 37°C by using a BioTek Synergy HT reader (BioTek Instruments Inc., Winooski, VT) at excitation/emission wavelength of 360 nm/460 nm.

### Ethics statement

All protocols for animal experiments were approved by the State Research Center for Applied Microbiology and Biotechnology Bioethics Committee (Permit No: VP-2016/4) and were performed in compliance with the NIH Animal Welfare Insurance #A5476-01 issued on 02/07/2007, and the European Union guidelines and regulations on handling, care and protection of Laboratory Animals (http://ec.europa.eu/environment/chemicals/lab_animals/home_en.htm).

### Animals

Seven week old male and female BALB/c mice (Lab Animals Breeding Center, Shemyakin and Ovchinnikov Institute of Bioorganic Chemistry, Russia) and four week old guinea pigs of both sexes (Lab Animals Breeding Center, Russian Academy of Medical Sciences, Stolbovaya, Moscow Region, Russia) were housed in polycarbonate cages, and maintained in light-controlled (lights on from 7:00 to 19:00) BSL3 room at the State Research Center for Applied Microbiology and Biotechnology. The temperature and the humidity of the animal room were maintained at 22°C ± 2°C and 50% ± 10%, respectively. Rodents were given tap water and mouse mixed fodder PK-120 or rabbit/guinea pig mixed fodder PK-122 (Laboratorkorm, Russia) *ad libitum* throughout the study. The number of animals used for the experiments were kept at the minimum dictated by the necessity, which was determined from the power analysis. The herds were divided into all groups randomly. In this study, we have used humane endpoints for the infected animals. According to the animal protocol, the mice and guinea pigs should be euthanized in the animal survival studies, when they became either of the following: lethargic, dehydrated, moribund, unable to rise, non-responsive to touch, or lost more than 10% body mass. Humane euthanasia using compressed CO_2_ gas followed by cervical dislocation has been used by well-trained individuals. We have monitored the health condition of the animals at least twice a day. There was no unexpected death during the entire set of experiments.

### Animal passage

To select for virulent *Y*. *pestis* subcultures, a group of two mice was challenged subcutaneously with 0.2 ml aliquots of each parent strain or their derivatives at a concentration of approximately 10^8^ cfu. The animals that were first to succumb to infection were subjected to necropsy and one bacteriologic loop of the brain tissue was suspended in 1 ml of 0.9% NaCl solution. In the second round of animal passage, two mice were challenged subcutaneously with 0.1 ml of this suspension containing approximately 200 cfu of *Y*. *pestis*. The cultures isolated from the animals that succumbed to early infection in the second passage were used in subsequent experiments. The animals that survived were humanely euthanized.

### Animal virulence challenge

Serial 10-fold dilutions of *Y*. *pestis* of two-day agar cultures of six strains grown at 28°C were administered subcutaneously in the interior thigh of 192 mice (10^3^ to 1 cfu, eight mice per one dose) and 216 guinea pigs (10^6^ to 10 cfu, six porpoises for one dose) or intradermally in the shaved lower back of 192 mice (10^3^ to 1 cfu, eight mice per one dose) (http://www.theodora.com/rodent_laboratory/injections.html). The actual number of bacteria present was determined by plating on agar medium. Humane endpoints were strictly observed. Animals that succumbed to infection were sacrificed and examined bacteriologically to verify that infection was the cause of death. The remaining animals were observed for 30 days. The animals that survived were humanely euthanized as described.

### Computational analysis of intrinsic disorder propensities of Pla isoforms

Amino acid sequences of the two forms of plasminogen activator Pla were analyzed for the effect of the I259T polymorphism on the intrinsic disorder propensities of related proteins. The intrinsic disorder of these Pla isoforms were evaluated by two disorder predictors, PONDR^®^ VSL2 [49], which is one of the more accurate stand-alone disorder predictors [49–51], and a metapredictor PONDR^®^ FIT [52], which is more accurate than each of its component predictors, PONDR^®^ VLXT [53], PONDR^®^ VSL2 [49], PONDR^®^ VL3 [54], FoldIndex [55], TOP-IDP-scale [56], and IUPred [57]. In addition to the two forms of Pla (UniProt ID: P17811), the intrinsic disorder of its homologues, outer membrane protease E (PgtE) from *Salmonella enterica* (UniProt ID: P06185) and OmpT protein from *Escherichia coli* O157:H7 (UniProt ID: P58603), were also evaluated by PONDR^®^ VSL2. Amino acid sequences of Pla and its bacterial homologues were aligned using the ClustalW2 multiple sequence alignment tool available at (http://www.ebi.ac.uk/Tools/msa/clustalw2/).

Since intrinsically disordered proteins or proteins with intrinsically disordered regions are frequently involved in protein-protein interactions and molecular recognitions [58–71] and undergo at least partial disorder-to-order transitions upon binding [60, 63, 71–78], these potential disorder-based binding sites can be identified by various computational means, such as the ANCHOR algorithm [79, 80]. Also, since intrinsic disorder is intimately linked to various posttranslational modifications (PTMs) [81–83], we used the ModPred tool [83] to evaluate how the I259T polymorphism affects the predisposition of the Pla C-terminal region for being posttranslationally modified.

### Statistical methods

The LD_50_ and a 95% confidence intervals were calculated using the Kärber method [84]. Mortality timeframes were recorded, and the mean life to death time span was compared by ANOVA. Comparison of the survival curves (a dose-dependent survival assay) was performed using Log-rank (Mantel-Cox) test. *P* values are indicated as *P≤0.05, **P≤0.01 and ***P≤0.001.

## 3. Results

### Distribution of Pla isoforms among natural isolates of *Y*. *pestis* of different origin

**Fig 1:**
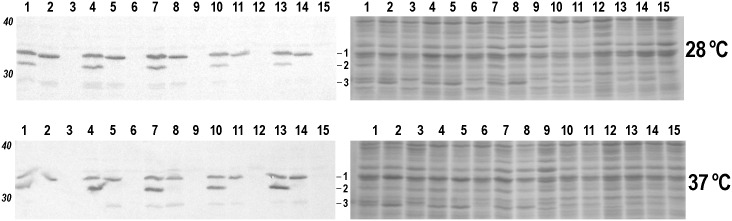
SDS-PAGE (right) and immunoblot analysis (left) of whole-cell lysates of the indicated by numbers *Y*. *pestis* strains with antibodies to Pla. Bacteria were cultured at the temperatures indicated on the right for each blot-gel pair. Molecular weight markers (Novex sharp protein standard, Life technologies) are shown in Italics on the left. Numbers and horizontal lines in the middle indicate Pla. The lower band represents the autoprocessed form of Pla. Track numbers correspond to: 1 –C-376pCD1^-^pkPEV, 2 –C-376pCD1^-^pkPI-3455, 3 –C-376pCD1^-^, 4 –C-585pCD1^-^pkPEV, 5 –C-585pCD1^-^pkPI-3455, 6 –C-585pCD1^-^, 7 –C-824pCD1^-^pkPEV, 8 –C-824pCD1^-^pkPI-3455, 9 –C-824pCD1^-^, 10 – 358pCD1^-^pPst^-^pkPEV, 11 – 358pCD1^-^pPst^-^pkPI-3455, 12 – 358pCD1^-^pPst^-^, 13 –KM217pkPEV, 14 –KM217pkPI-3455, 15 –KM 217.

Amplification and sequencing of *pla* genes from 118 *Y*. *pestis* subsp. *microtus* isolates revealed the absence of this gene in the strains belonging to bv. caucasica. All remaining isolates of subsp. *microtus* contained the ancestral Pla isoform (I259). This observation was confirmed by immunoblot analysis of whole-cell lysates of the *Y*. *pestis* strains probed with anti-Pla MAb (Fig 1). All isolates of the subsp. *microtus* displayed on immunoblot a 36.9 kDa single band corresponding to the native form of Pla (Fig 1B, lines 1–5), while the strains of the main subsp. *pestis* had an additional band of 34.1 kDa corresponding to the autoprocessed form of Pla (Fig 1B, lines 6, 7, 9). It is necessary to note that the Pla peptide had an atypical mobility on the gel, since the molecular weight of the native and autoprocessed forms of the Pla deduced from their amino acid sequences corresponded to those of 32.6 and 29.6 kDa, respectively. Introduction of the plasmid encoding the “modern” isoform of Pla (T259) or its “ancestral” isoform Pla (I259) into pPst^-^ strain C-376pCD1^-^ of bv. caucasica revealed that the Pla isoforms were expressed and autoprocessed in this strain identically to that observed for the natural isolates (Fig 1B, lines 10, 11). No significant difference could be detected between the bands suggesting that equal numbers of bacteria produce equal amounts of Pla protein.

### Evaluation of enzymatic activity of Pla isoforms

Coagulase and fibrinolytic activities of Pla isoforms were determined in five isogenic sets of strains belonging to both *Y*. *pestis* subspecies, such as *pestis* and *microtus* (Table 2). Introduction of plasmid containing the *pla* gene into pPst^-^ strains resulted in appearance of both activities. The “modern” Pla isoform T259 of the epidemic strains was visibly more effective in both coagulase and fibrinolytic activities than the “ancestral” isoform I259 found in the endemic strains of subs. *microtus*.

**Table 2:** Pla isoforms enzymatic activities.

Strain	Pla isoform	coagulase	fibrinolytic
358pCD1^-^pPst^-^	–	–	–
358pCD1^-^pPst^-^pkPI-3455	I259	+	+
358pCD1^-^pPst^-^pkPEV	T259	++	++
KM217	–	–	–
KM217pkPI-3455	I259	+	+
KM217pkPEV	T259	++	++
C-376pCD1^-^	–	–	–
C-376pCD1^-^pkPI-3455	I259	+	+
C-376pCD1^-^pkPEV	T259	++	++
C-585pCD1^-^	–	–	–
C-585pCD1^-^pkPI-3455	I259	+	+
C-585pCD1^-^pkPEV	T259	++	++
C-824pCD1^-^	–	–	–
C-824pCD1^-^pkPI-3455	I259	+	+
C-824pCD1^-^pkPEV	T259	++	++

**Fig 2:**
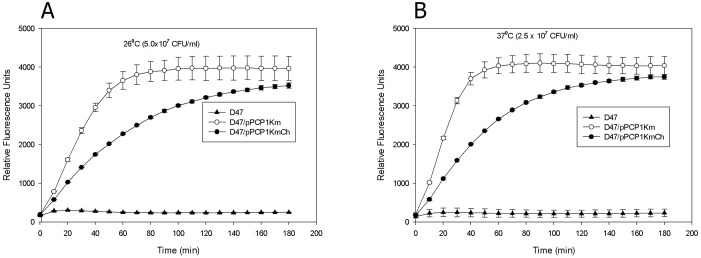
Functional activity of two isoforms of Pla protease determined by the fluorometric assay with the Pla substrate DABCYL-Arg-Arg-Ile-Asn-Arg-Glu (EDANS)-NH_2_. The kinetics of the substrate cleavage was measured in quadruplicates for *Y*. *pestis* strains KIM D47, KIM D47/pPCP1Km, and KIM D47/pPCP1KmCh grown at either 26°C (A) or 37°C (B). The graphs were plotted as arithmetic means ± standard deviations. Statistical analysis of Pla activity data was done by one-way ANOVA with a Bonferroni *post hoc* test. The differences between the groups expressing the Pla (T259) and (I259) isoforms for both growth temperatures were statistically significant (*P* < 0.01).

To further compare the activity of the Pla isoforms, we used a microplate assay with the fluorescently labeled hexapeptide substrate DABCYL-Arg-Arg-Ile-Asn-Arg-Glu (EDANS)-NH_2._ The Pla protease directly cleaves the substrate between two basic amino acids, such as arginines [48]. The kinetics of the substrate cleavage revealed that the subsp. *pestis* Pla isoform T259 was significantly more active (*P* < 0.01) than the subsp. *microtus* Pla isoform I259. This difference was observed for the Pla protease displayed on the surface of bacteria grown either at 26°C or 37°C (Fig 2).

### Intrinsic disorder propensity

**Fig 3:**
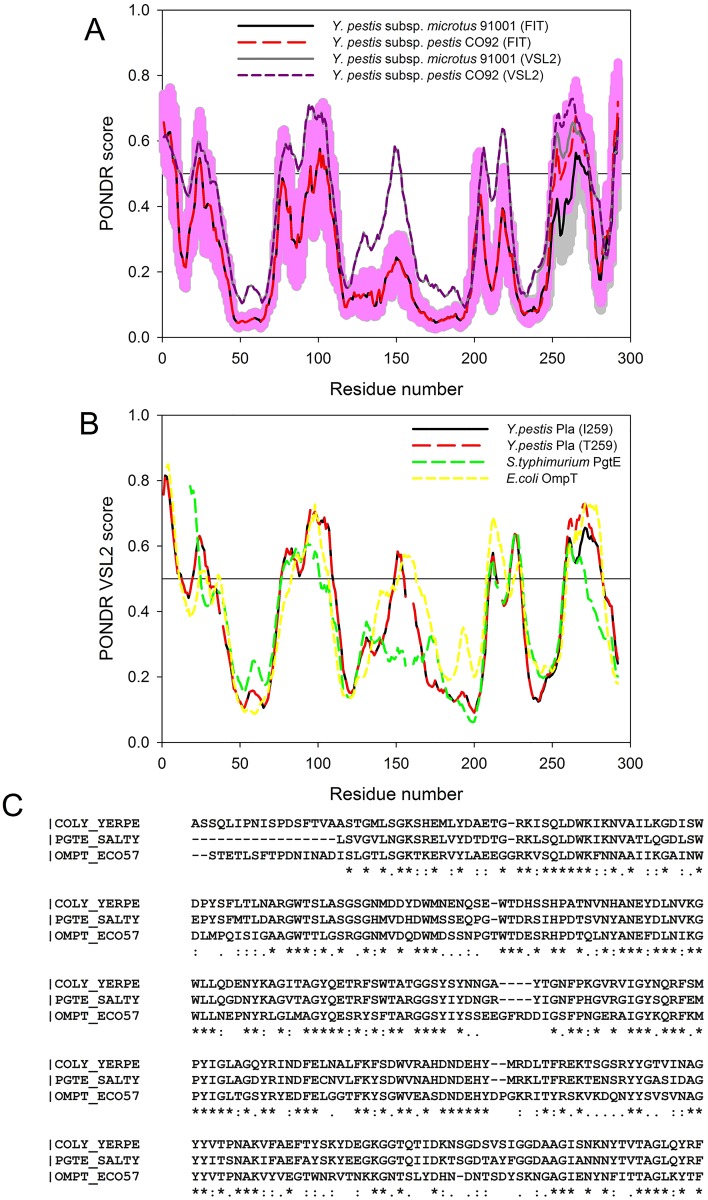
Predicted intrinsic disorder propensities of the two *Y*. *pestis* Pla isoforms. A. Evaluating predicted intrinsic disorder propensities of the two Pla isoforms [*Y*. *pestis* subsp. *microtus* 91001 (solid black and gray lines) and *Y*. *pestis* subsp. *pestis* CO92 (dashed red and dark red lines)] by PONDR^®^ VSL2 (solid gray and dashed dark red lines) and POND-FIT (solid black and dashed red lines). Disorder scores above 0.5 correspond to the residues/regions predicted to be intrinsically disordered. Colored (light pink and light gray) shades around the corresponding PONDR-FIT curves represent distributions of errors in evaluation of disorder propensity. B. Comparison of the disorder propensities of the two forms of Pla (UniProt ID: P17811), with those of its homologues, outer membrane protease E (PgtE) from *S*. *enterica* (UniProt ID: P06185) and OmpT protein from *E*. *coli* O157:H7 (UniProt ID: P58603). The corresponding disorder profiles were obtained by PONDR^®^ VSL2. Sequences were aligned using the ClustalW2 multiple sequence alignment tool and the results of the alignment are shown in panel C.

Fig 3 shows that intrinsic disorder propensity and potential disorder-based functionality was moderately affected by the I259T polymorphism. In fact, in the T259 form of Pla, C-terminal tail is more disordered, has more potential sites of posttranslational modifications (T259 is a novel phosphorylation site induced by the I259T mutation), and shows a bit higher propensity for interaction with other proteins as judged by the local increase in the ANCHOR scores in the C-terminal part of the protein (data not shown). To understand if peculiarities of the disorder distribution are conserved within the members of the omptin family, we compared the disorder profiles of the two forms of Pla, and its two homologues, the outer membrane protease E (PgtE) from *S*. *enterica*, and the OmpT protein from *E*. *coli* O157:H7. Results of this analysis are shown in Fig 3B which shows remarkable similarity between these three proteins in respect of their disorder profiles. This similarity of disorder distributions is particularly appealing in light of rather limited sequence identity of these three proteins (*Y*. *pestis* protein is 76.4 and 51.0% identical to proteins from *S*. *enterica* and *E*.*coli*, see Fig 3C). The high level of conservation in the disorder-related features suggests that the intrinsically disordered and flexible regions might play important functional roles in members of the omptin family.

### Virulence study of *Y*. *pestis* expressing different isoforms of Pla in a bubonic plague model

**Fig 4:**
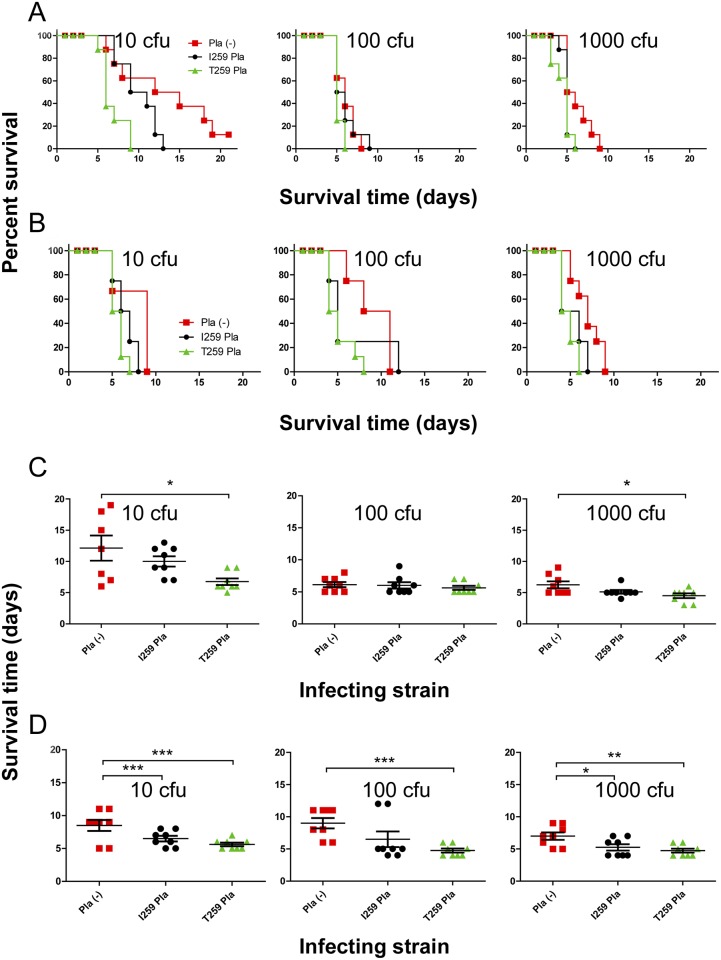
Kinetics of survival in subcutaneously inoculated mice. (A and B) The dose-dependent survival assays and (C, D) mean time to death post infection of mice (*n* = 8 in a group) inoculated with three different doses of the strains (A, C) C-267 and (B, D) 231 isogenic derivatives (Pla (-)) deficient in Pla or producing its I259 (pKP3455) or T259 (pKPEV) isoform. *, *P*<0.05, **, *P*<0.01, ***, *P*<0.001. The planned injection dose of 100 cfu was actually equal to 98 (C-267), 115 (C-267pKP3455), 123 (C-267pKPEV), 103 (231pPst^-^), 95 (231pPst^-^pKP3455) or 75 (231pPst^-^pKPEV) cfu.

**Fig 5:**
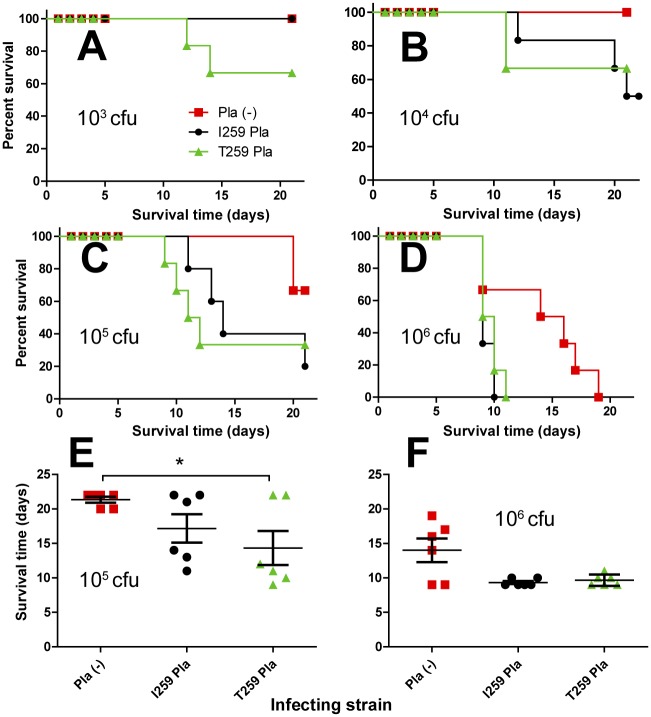
Kinetics of survival in subcutaneously inoculated guinea pigs. (A, B, C, D) The dose-dependent survival assays and (E and F) mean time to death post infection of guinea pigs (*n* = 6 in a group per inoculum) inoculated with four different doses of the strain 231 isogenic derivatives (Pla (-)) deficient in Pla or producing its I259 or T259 isoform. 10^3^ (A), 10^4^ (B), 10^5^(C, E), and 10^6^ (D, F) cfu of *Y*. *pestis* variants, respectively. The planned injection dose of 100 cfu was actually equal to 103 (231pPst^-^), 95 (231pPst^-^pKP3455) or 75 (231pPst^-^pKPEV) cfu. Compared by ANOVA. *P<0.05.

We did not observe a statistically significant difference between the groups of mice and guinea pigs, which received subcutaneous challenge with *Y*. *pestis* expressing either isoform of the Pla with regards to the LD_50_ values (Table 3) or survival curves (Figs 4 and 5). However, in mice the survival curves indicate that T259 isoform was associated with the deaths in the earliest period after infection. Indeed, the dose-dependent survival assay shows that the mean times to death significantly differ as a rule between T259 Pla^+^ and Pla^−^isogenic strains, while I259 Pla^+^ strains occupy an intermediate position differing insignificantly from the first or the last in the case of C-267 background. In the case of 231-related isogenic strains, we can see, in addition, a significant difference between Pla^−^and I259 Pla^+^ variants.

**Table 3:** Subcutaneous virulence of *Y*. *pestis* strains differing in ability to produce Pla isoforms.

*Y*. *pestis* strain	Pla isoform	LD_50_ (CFU)* for	
		Mice	Guinea pigs
subsp. *microtus* bv. caucasica			
C-267	–	1 (1–6)	> 10^6^
C-267pkPI-3455	I259	7 (2–27)	> 10^6^
C-267pkPEV	T259	11 (3–42)	> 10^6^
subsp. *pestis* bv. *pestis*			
231pPst^-^	–	18 (4–71)	1.5 × 10^5^ (3.7 × 10^4^−9.3 × 10^5^)
231pPst^-^pkPI-3455	I259	18 (4–71)	1.5 × 10^4^ (3.7 × 10^3^−5.8 × 10^4^)
231pPst^-^pkPEV	T259	3 (1–13)	1.1 × 10^4^ (2.7 × 10^3^−4.4 × 10^4^)

*Values in parentheses represent the 95% confidence interval of the LD_50_.

The Pla protease did not increase virulence for guinea pigs of *Y*. *pestis* C-267 representing subsp. *microtus* bv. caucasica that are naturally pPst^-^: the C-267 displaying Pla on its surface was still avirulent in this animal model as was the parent strain (Table 3). The results of comparative dose-dependent survival assay in guinea pigs infected with the strains from the isogenic set on the background of initially virulent for them subsp. *pestis* strain 231 are shown in Fig 5. Relative location of survival curves of guinea pigs infected with this group of strains follows the pattern typical for mice, but significant differences were shown only for the pair, T259 Pla^+^ and Pla^−^, infected with 10^5^ cfu (Fig 5).

**Fig 6:**
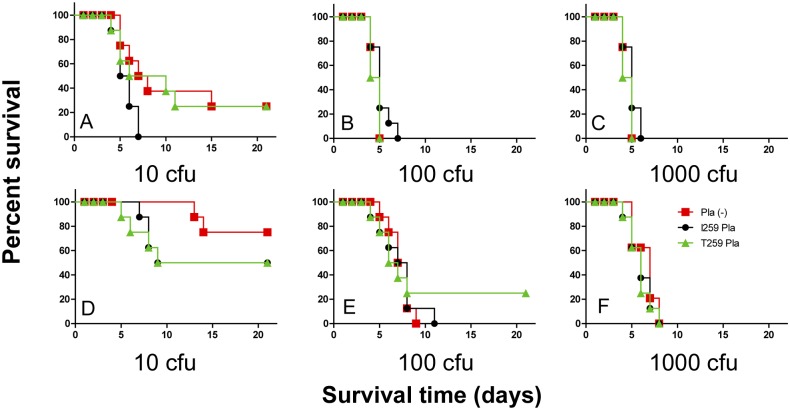
Comparison of survival in mice subcutaneously or intradermally inoculated with the strain C-267. The dose-dependent survival assays post infection of mice (*n* = 8 in a group per inoculum) inoculated either subcutaneously (upper panels), or intradermally (lower panels) using three different doses of 10 (A, D), 100 (B, E), or 1000 (C, F) cfu of the strain C-267 isogenic derivatives (Pla (-)) deficient in Pla (squares) or producing its I259 (circles) or T259 (triangles) isoform. The planned injection dose of 100 cfu was actually equal to 53 (C-267), 58 (C-267pKP3455), 74 (C-267pKPEV) cfu.

**Fig 7:**
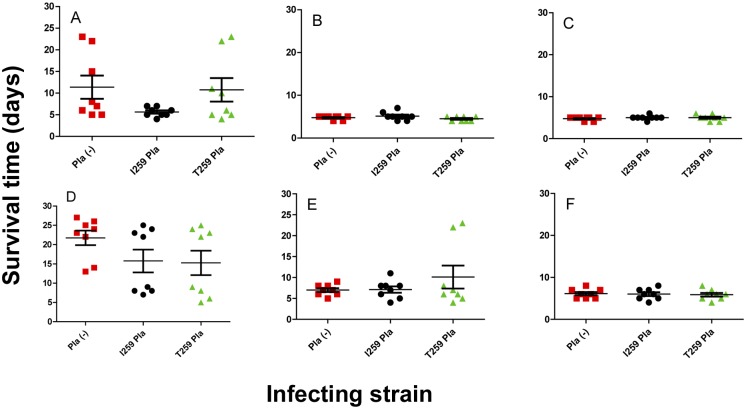
Comparison of mean time to death post infection in mice subcutaneously or intradermally inoculated with the C-267. The mean time to death post infection of mice (*n* = 8 in a group per inoculum) inoculated either subcutaneously (upper panels), or intradermally (lower panels) using three different doses of 10 (A, D), 100 (B, E), or 1000 (C, F) cfu of the strain C-267 isogenic derivatives (Pla (-)) deficient in Pla (circles) or producing its I259 (triangles) or T259 (squares) isoform. The planned injection dose of 100 cfu was actually equal to 53 (C-267), 58 (C-267pKP3455), 74 (C-267pKPEV) cfu.

**Fig 8:**
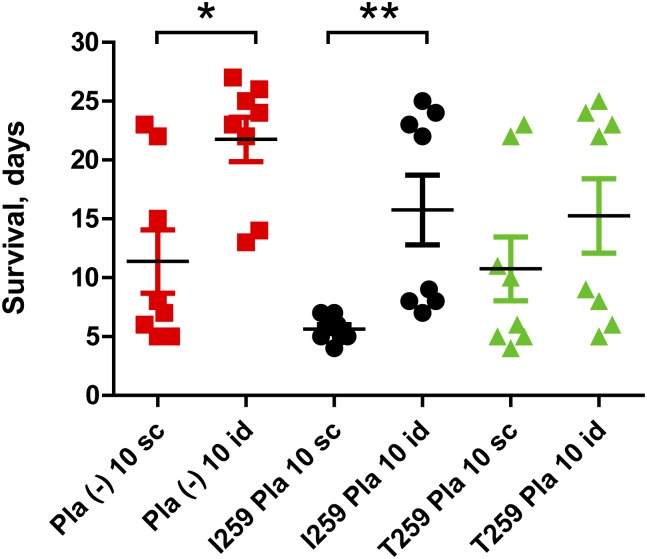
Comparison of different routes of infection of mice with the same isogenic strains, C-267 derivatives. *, *P*<0.05; **, *P*<0.01. Mann-Whitney test. 10 sc/10 id, 10 cfu via subcutaneous/intradermal route. The actual injection doses were equal to 5 (C-267), 6 (C-267pKP3455), 7 (C-267pKPEV) cfu per mouse.

In addition, we attempted to assess variations in virulence between isogenic strains, which differ in production of the Pla isoform by using the intradermal route of inoculation in mice. This route of infection is believed to be the most appropriate in modeling the flea bites [85]. The intradermal infection of mice provided the results (Table 4; Figs 6 and 7) similar to those obtained with subcutaneous route. Nevertheless, in this experiment, we did not observe differences not only between the LD_50_ values and survival curves, but also between the mean times to death post infection. The only significant differences were seen in the mean times to death in the groups of mice infected with the lowest doses of approximately 10 cfu (Fig 8). The survival time of animals infected intradermally with all three strain variants significantly increased in comparison with that of the subcutaneous route.

**Table 4:** Comparative virulence of *Y*. *pestis* C-267 derivatives differing in ability to produce Pla isoforms in the case of subcutaneous or intradermal infection.

*Y*. *pestis* subsp. *microtus* bv. caucasica strain	Pla isoform	LD_50_ (CFU)* for route of infection	
		subcutaneous	intradermal
C-267	–	3 (1–12)	5 (1–21)
C-267pkPI-3455	I259	2 (1–7)	6 (1–23)
C-267pkPEV	T259	4 (1–17)	13 (3–52)

*Values in parentheses represent the 95% confidence interval of the LD_50_.

In infections with lower doses of bacteria, we observed two peaks in mouse (Figs 4, 7 and 8) and guinea pig (Fig 5) mortality, separated by 10–20 days. This phenotype was observed regardless of the strain of bacteria used or the sex of the animals.

Postmortem pathological pattern in animals did not reveal difference between the groups that received *Y*. *pestis* with or without different Pla isoforms, while the time of appearance of typical signs of plague was consistent with the life expectancy. Inflammation and swelling of the regional draining lymph nodes in the area of inoculation of the bacteria characteristic of bubonic plague [16] was observed in all dead animals.

## 4. Discussion

A complete genome sequence of *Y*. *pestis* subsp. *microtus* strain 91001 [86] and consequent release of a few genomes of the endemic strains with limited virulence revealed the presence of a single nucleotide polymorphism within the *pla* gene resulting in Pla polymorphism at position 259 (I259T). Our study on distribution of *pla* alleles in 118 isolates of *Y*. *pestis* subsp. *microtus* showed, for the first time, that the isoform I259 Pla was found exclusively in the endemic strains, providing a convincing evidence of more ancestral origin of the I259 Pla isoform. In all of the subsp. *microtus* representatives under the study belonging to the SNP types of 0.PE cluster [37, 43, 86, 87] this gene was either absent (bv. caucasica (0.PE2)) or was in its ancestral I259 isoform (bvv. talassica (0.PE4), hissarica (0.PE4), altaica (0.PE4), qinghaiensis (0.PE4ab), xilingolensis (0.PE4cd) и ulegeica (0.PE5)). This data is a strong argument in favor of the importance of the I259T substitution during the *Y*. *pestis* transition from “selective” to “universal” virulence. However, this argument is contradicted by rare reports on individual I259 or even Pla^−^strains from the group 0.PE (bvv. caucasica, ulegeica [1], 0.PE4 and 0.PE7 groups [88]) that are highly virulent for guinea pigs and can cause disease in humans. Also, the discovery of the oldest 0.PE7 strains and even DNA from Bronze Age *Y*. *pestis* harboring the *pla* gene [89] put doubt in the long-lived hypothesis that the acquisition of the pPst plasmid occurred only after branching of bv. caucasica. Most likely the progenitor of this biovar had lost the pPst plasmid just before or immediately after diverging from the mainstream.

Analysis of the expected effects of the I259T polymorphism on the intrinsic disorder propensity of Pla, its ability to be engaged in disorder-based protein-protein interactions and to have potential PTM sites revealed that mutating isoleucine at position 259 slightly increases the intrinsic disorder propensity of the C-terminal tail of Pla and makes this protein slightly more prone for disorder-based protein-protein interactions. Also, this mutation affects the predisposition of Pla for PTMs since it introduces a novel phosphorylation site. All this suggests that the T259 Pla is expected to be functionally more active than the I259 version of this protein.

Curiously, there is a good correlation between the results of the disorder predictions and the actual structural information available for the I259T form of the *Y*. *pestis* plasminogen activator Pla [90]. In fact, according to the crystal structure of this protein, Pla has five extracellular loops (L1, residues 26–39; L2, residues 78–107; L3, residues 143–173; L4, residues 201–220; and L5, residues 254–281, that contains the I259T polymorphism). Fig 2A shows that these extracellular loops correspond to or at least partially overlap with the regions of predicted disorder (i.e., regions with the disorder score >0.5) or regions of increased flexibility (i.e., regions with the disorder score >0.25). The fact that the I259T mutation significantly increases the local disorder propensity of loop 5 (see Fig 3A) suggests that this region could be rather flexible in the T259 form of Pla. This hypothesis is supported by the results of structural analysis, since it has been reported that the significant portion of the extracellular loop L5 (namely, residues 253–268) is not visible in the crystal structure of the mature plasminogen activator Pla (PDB ID: 2X55) due to the high conformational flexibility of this region and potential autocatalytic activity of Pla at residue K261 [90]. In fact, this region was present in the crystal structure of the inactive D86A mutant in a form of a β-hairpin opposing the β-hairpin found in a loop L3 (PDB ID: 2X4M) [90].

It was also pointed out that the plasminogen activator Pla from *Y*. *pestis* belongs to the omptin family of the enterobacterial surface proteases (serine proteases that lack the classical consensus sequences typical for serine proteases) and shows significant sequence similarity with other members of this family [18]). Importantly, the β-barrel fold found in the outer membrane proteins (such as the OmpT protein of *E*.*coli*) is a stable entity that shows remarkable resistance towards the changes and mobility of the surface-exposed loops [91–93]. In fact, the structural comparison of Pla and OmpT indicated that both structures were very similar within their membrane-embedded parts that constitute the aforementioned β-barrel fold and contain the active sites, but were characterized by the pronounced differences in the extracellular loops, most notably in loops L3, L4, and L5 [90]. The surface loops were assumed to play a role in controlling the substrate specificity of omptins [18]. In agreement with this idea, the *E*.*coli* OmpT was converted into the efficient plasminogen activator by making its loops L3 and L4 (which form the entrance to the active site and are very likely to be involved in substrate binding) similar to those of Pla [18]. Furthermore, this analysis of the Pla-OmpT hybrids produced strong support to the role of the loop L5 in controlling the substrate specificity of omptins. In fact, substitution of the L5-containing C-terminus of Pla with the corresponding region of OmpT resulted in almost complete loss of the plasminogen activating capacity of the corresponding hybrid. The reverse substitution in OmpT (where the OmpT C-terminal region was substituted to the analogous region of Pla) generated a protein possessing noticeable plasminogen activating potential as judged by the fast and efficient degradation of the S-2251 chromogenic substrate of the plasminogen [18].

Additional confirmation of the potential functional importance of disordered/flexible extracellular loops L1-L5 can be found in very close similarity of disorder profiles obtained for *Y*. *pestis* Pla and its homologues from *S*. *enterica* and *E*.*coli*, that share rather limited sequence identity. It is logical to hypothesize that the evolutionary conserved peculiarities of the disorder distribution within the amino acid sequences of the members of the omptin family might be related to the functional utilization of these disordered/flexible regions.

Overall, combining these earlier observations with the results of our analyses on the influence of the I259T substitution on intrinsic disorder propensities and functions of the *Y*. *pestis* Pla provides a mechanistic explanation for the increased protease activity of Pla isoform typical of all highly virulent for humans strains of *Y*. *pestis* subsp. *pestis*.

In this study, we made a comparative analysis of the enzymatic activities of the two Pla isoforms by using a semiquantitative approach on evaluation of coagulase and fibrinolytic activities in human plasma, as well as quantified a direct cleavage of the fluorescent Pla peptide substrate. These methods convincingly demonstrated an increased activity of the T259 isoform of Pla that is in agreement with the previously obtained results [38, 39].

To assess the influence of the Pla isoform on virulence of *Y*. *pestis*, we used strains with two genetic backgrounds, such as 231 of subsp. *pestis* bv. antiqua (0.ANT) cured from the small plasmid (231pPst^-^) and C-267 subsp. *microtus* bv. caucasica (0.PE2) naturally free from the pPst plasmid. After introduction to these strains of the pPst plasmid encoding for one of the two Pla isoforms, virulence was tested in a bubonic plague model in mice and guinea pigs. We have not found a statistically significant difference in subcutaneous LD_50_ values and survival curves between the Pla^-^ strains and their derivatives expressing the Pla isoforms in both animal models. Moreover, the expression of the Pla in *Y*. *pestis* C-267 did not change the virulence of this strain for guinea pigs, indicating that the failure of the strains of bv. caucasica to cause disease in this animal model (Anisimov et al., 2004) is not related to the presence of the Pla protease. Similarly, to previously reported data with *Y*. *pseudotubeculosis* [94], the expression of Pla in *Y*. *pestis* C-267 did not change the LD_50_ values of this strain in a murine model of bubonic plague; however, it is necessary to note that the parent strain C-267 was quite virulent for BABL/c mice with the LD_50_ ranging within 1 to 6 cfu (Table 3). Thus, a possible increase in virulence might be difficult to judge. Generally, the subcutaneous LD_50_ values for mice infected with subsp. *microtus* bv. caucasica isolates did not exceed 15 cfu regardless of the reporting laboratory [1, 13, 33].

Similar data generated in different laboratories for subcutaneous or intradermal inoculation of Pla^−^strains of the main subspecies *pestis* differed from each other while being similar for Pla^+^ strains [44, 95] or being four to six logs greater than that of the wild type [11, 13, 16]. These differences can be explained by a different location of the strains on the phylogenetic tree of the plague pathogen. We used the strain of the main subspecies 231 that was located on the 0 branch just after subsp. *microtus* isolates, while the strains studied by others that showed the dramatic dependence of subcutaneous virulence upon the Pla presence were assigned to the 1^st^ or 2^nd^ branches. It cannot be excluded that, in the course of adaptation of *Y*. *pestis* to the dissimilar variants of its new ecological niche, selection of the two clonal clusters differing in Pla-dependence of their virulence diverged into endemic and pandemic strains. The molecular basis of this difference requires further investigation.

Our data differs from the recent finding by Zimbler *et al*. [39] who investigated virulence of *Y*. *pestis* variants obtained by the introduction of Pla into Pestoides F, another bv. caucasica strain. Their observations were based on dissemination of isogenic strains from the site of subcutaneous inoculation to lymph nodes and spleens, and showed that the I259T modification of Pla was necessary for *Y*. *pestis* to cause a transient systemic infection. Our studies complement rather than contradict their data. Our T259-isoform-dependent reduction in the time to death of infected animals is consistent with that described by Zimbler *et al*. [39] on accelerated dissemination of T259-Pla^+^ bacteria resulting in “more rapidly progressing forms of disease caused by *Y*. *pestis*, rather than increased lethality”.

It is well-known that a rapid course and a high mortality of infection combined with its low transmissibility are often limiting the epizootic process [96, 97]. The problem of low transmissibility was solved by the plague pathogen 950–1700 years ago by acquisition of the *ymt* gene [89] coding for a phospholipase D, protecting bacteria from the vector, flea, digestive tract antimicrobial defenses, thus ensuring efficient transmission [98]. According to R.R. Brubaker [99], in contrast to chronic infections caused by the enteropathogenic yersiniae, “plague is manifested as an acute, intransigent, and lethal disease since the infective agent requires the death of its host in order to assure perpetuation via transfer by the disenfranchised vector. This strategy is dependent upon mounting an immediate overwhelming attack on the host before its immune system becomes capable of providing significant defense”.

Recently Gonzalez *et al*. published the study [100] suggesting that *Y*. *pestis* infection via the subcutaneous route may not distinguish between small differences in virulence, and that the intradermal route [85] is a better model for comparative study of bubonic plague caused by the Δ*rovA* mutant and its parent wild-type strain [100]. Our data suggest that, for comparison of Pla isoforms in virulence studies, both routes are equally informative.

The presence of two peaks of mortality in some animal groups is unclear, but may be explained by the presence of two subpopulations in each rodent species. Both male and female mice demonstrated the same bimodal survival curves with two peaks of mortality. Therefore, we do not believe that the two different peaks were dependent on the animal gender. The nature of this dissimilarity not only within outbreed guinea pigs but also within inbreed mice needs further investigations.

## Conclusions

We found no significant difference in LD_50_ values and dose-dependent survival assays between the strains with the I259 and T259 Pla isoforms by using either subcutaneous route of challenge of mice and guinea pigs, or intradermal challenge of mice. The significant difference could be seen in mean time to death post infection between the Pla^−^strains and their T259 Pla^+^ variants in both sets of isogenic strains. Survival curves of the I259 Pla^+^ strains were located in the intermediate position between them, but significant difference in mean time to death post infection between the Pla^−^strains and their I259 Pla^+^ variants could be seen only in the isogenic set of subsp. *pestis* strains. This suggests the presence of unidentified mutation(s) responsible for a better adaptation of bacteria to the most effective expression of the gene encoding T259 Pla in the strains from the younger branches of the *Y*. *pestis* phylogenetic tree. Most likely T259 Pla-dependent acceleration of this lethal infection was the final step in the transformation of the highly virulent but endemic pathogen into the pandemic strain whose descendants caused three devastating pandemics.
